# Retraction: Anti *H. pylori*, anti-secretory and gastroprotective effects of *Thymus vulgaris* on ethanol-induced gastric ulcer in Sprague Dawley rats

**DOI:** 10.1371/journal.pone.0326248

**Published:** 2025-06-12

**Authors:** The PLOS One Editors

The *PLOS One* Editors retract this article [1] due to concerns about:

- Reliance on retracted work cited in References 1, 6, 8, 12, 14, 20, 21, 26, 45, 46, 50, and 53.
- The reliability of the reported results and conclusions.

The author did not agree with the retraction.
